# Extraction of a metallic susceptor after accidental ingestion of the heated tobaccostick TEREA™: a case report

**DOI:** 10.1186/s12887-023-04285-7

**Published:** 2023-09-09

**Authors:** Koki Higashi, Yuhki Koike, Yuki Sato, Shinji Yamashita, Yuka Nagano, Tadanobu Shimura, Takahito Kitajima, Kohei Matsushita, Kazuki Yokota, Keishiro Amano, Yoshinaga Okugawa, Yuji Toiyama

**Affiliations:** 1 Department of Gastrointestinal and Pediatric Surgery, Mie University Graduate School of Medicine, Tsu, Mie Japan; 2 Department of Pediatrics, Mie University Graduate School of Medicine, Tsu, Mie Japan

**Keywords:** Heated tobacco products, Ingestion, Pediatric, Endoscopy

## Abstract

**Background:**

Tobacco ingestion is widely known to cause nicotine toxicity, which may result in severe symptoms. Two heated tobacco sticks, called TEREA™ and SENTIA™, were launched in 2021 by Philip Morris International (New York, NY, USA), and their ingestion is associated with a risk of bowel injury because they contain a partially pointed metallic susceptor. However, this risk is not well known to the general public or healthcare providers. To increase awareness of this risk, we herein report a case involving extraction of a metallic susceptor after ingestion of the heated tobacco stick TEREA™.

**Case presentation:**

A 7-month-old girl presented to the emergency department of a nearby hospital because she was suspected to have accidentally swallowed heated tobacco. Although she presented with no symptoms related to nicotine poisoning, abdominal X-ray examination revealed a metal object in her stomach. According to a statement released by the Japan Poison Information Center, the TEREA™ heated tobacco stick contains a metallic susceptor with a rectangular shape and sharp corners. The patient was transferred to our department because of the risk of bowel injury, and upper gastrointestinal endoscopy was performed. No cigarettes were found by endoscopic observation; however, a metallic susceptor was located in the second part of the duodenum. We grasped it with biopsy forceps and carefully removed it using an endoscope with a cap attached to the tip. The post-endoscopic course was uneventful.

**Conclusions:**

Some patients who ingest heated tobacco sticks might be exposed not only to the effects of nicotine but also to physical damage caused by a metallic susceptor. Infants and toddlers especially could swallow these sticks, therefore tobacco companies need to make the problem more public. Clinicians also should alert the problem, and pay attention to this risk in the clinical setting.

## Background

Foreign body ingestion is a common problem among children. Some ingested foreign bodies can cause serious complications because of their properties. Tobacco ingestion may induce nicotine poisoning, and even the ingestion of low doses of nicotine can result in systemic toxic effects. Nicotine exposure leads to stimulate the sympathetic nervous system because of its bindings at nicotinic acetylcholine receptors and it causes parasympathetic stimulation and neuromuscular blockade with high doses [1]. Nicotine poisoning results in initially a number of symptoms such as nausea, tachycardia, salivation, abdominal pain, hypertension, increased bronchial secretions, anxiety, and seizures [2, 3]. Later, inhibit symptoms may appear like bradycardia, low blood pressure, dyspnea, coma, and paralysis [2]. Although this is well known among the general public, most people are unaware that some newly heated tobacco sticks contain metallic objects that can lead to bowel injury if ingested. We herein describe a case of endoscopic removal of a metallic susceptor reportedly contained in some heated tobacco sticks.

## Case presentation

**Fig. 1:**
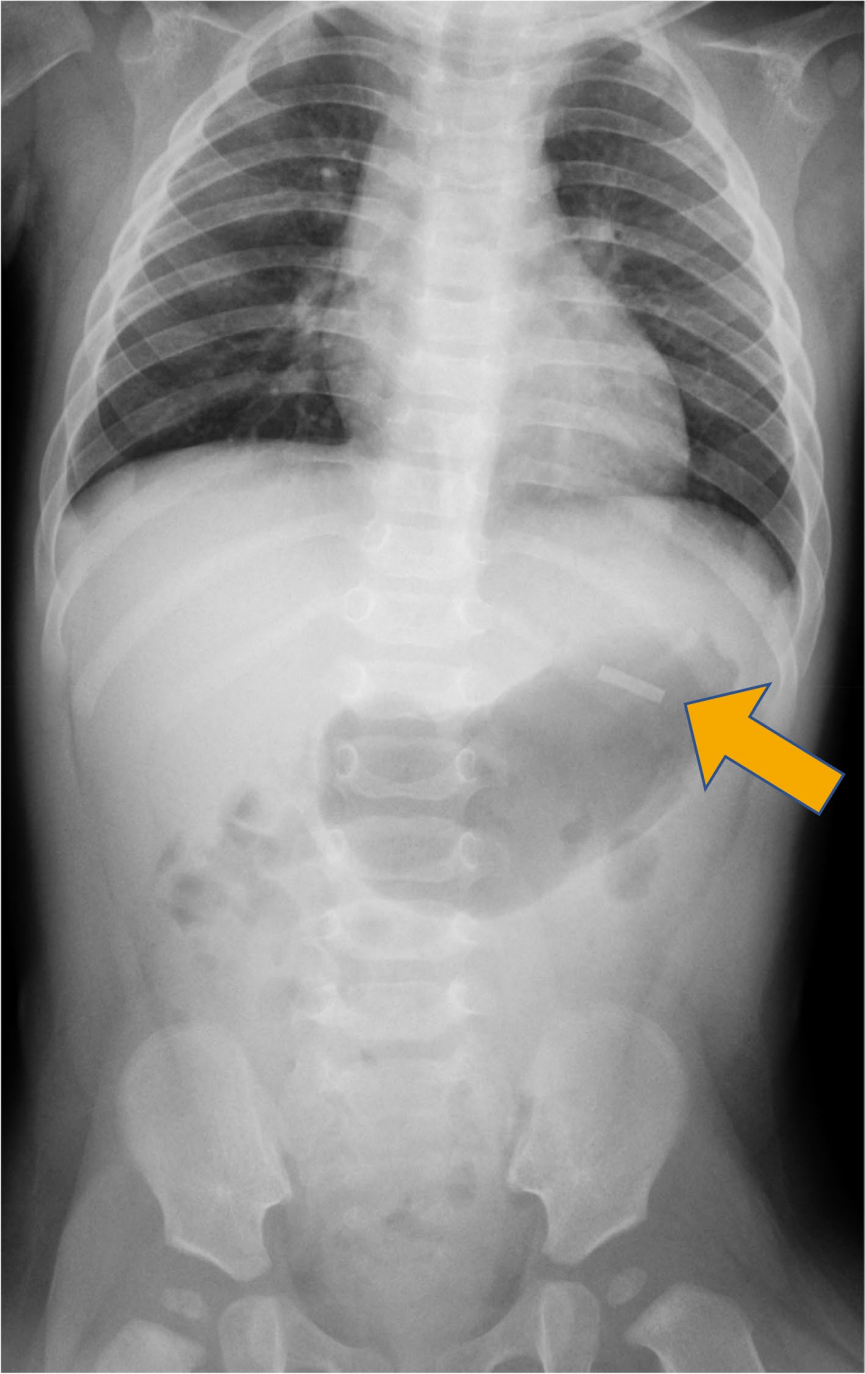
Initial abdominal X-ray. The X-ray revealed a metallic foreign body in the stomach (arrow)

A 7-month-old girl presented to the emergency department of a nearby hospital because she was suspected to have accidentally swallowed heated tobacco. She had no symptoms suggestive of nicotine poisoning. Physical examination was unremarkable, and all laboratory values were within normal limits. An initial abdominal X-ray revealed a metallic foreign body within her stomach (Fig. 1). Because of the risk of bowel injury, she was transferred to our department for further evaluation and treatment. According to a statement released by the Japan Poison Information Center, a heated tobacco stick called TEREA™ (Philip Morris International, New York, NY, USA) contains a metallic susceptor with a rectangular shape and sharp corners. A secondary abdominal X-ray showed that the foreign body was still present in her stomach; therefore, upper gastrointestinal endoscopy was performed for extraction of the object.

**Fig. 2:**
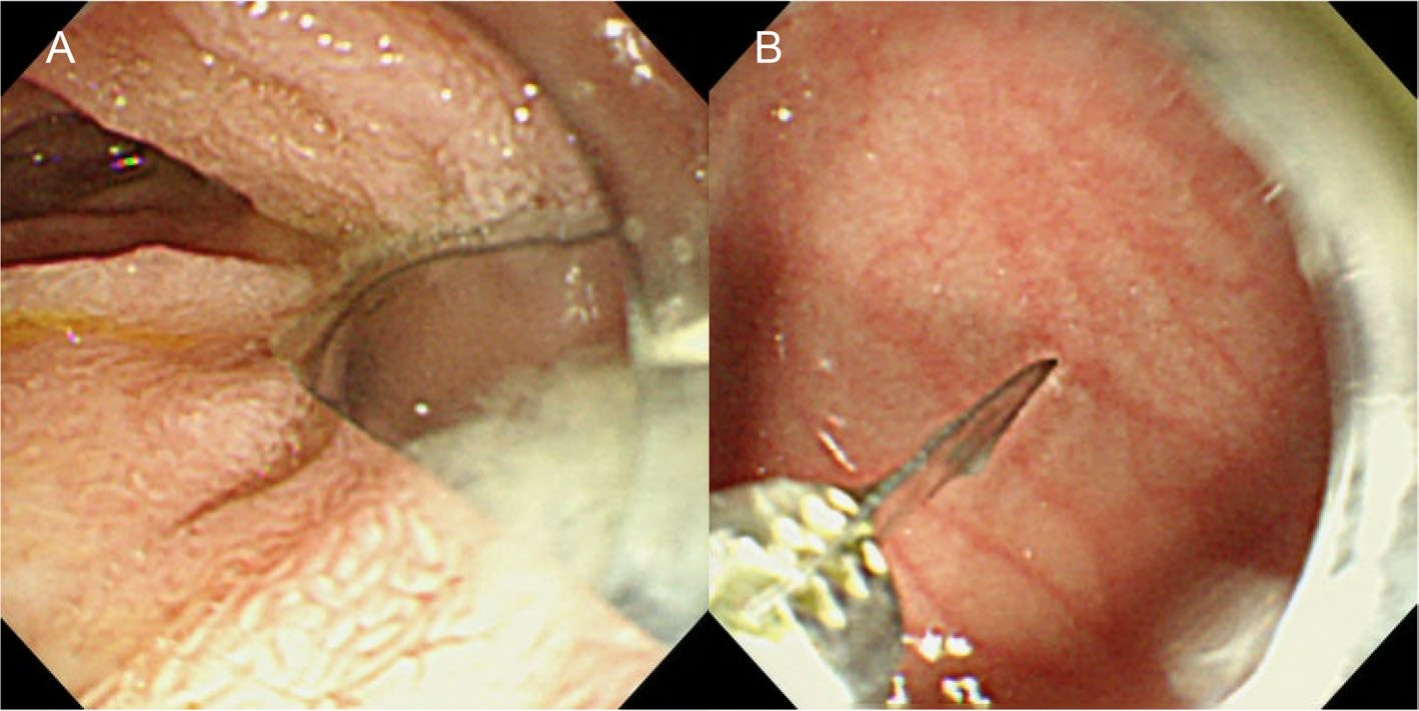
Endoscopic images. **A** A metallic susceptor located in the duodenum. **B** Endoscopic removal of the metallic susceptor with biopsy forceps

The endoscopic examination revealed no cigarettes; only a metallic susceptor was located in the second part of the duodenum (Fig. 2A). We grasped it with biopsy forceps and carefully removed it using an endoscope with a cap attached to the tip (Fig. 2B). The removal procedure resulted in only slight esophageal mucosal abrasion. The post-endoscopic course was uneventful.

## Discussion

Accidental ingestion of cigarettes and other tobacco products can sometimes lead to nicotine toxicity. Nicotine poisoning is well documented in the pediatric population. Children aged 6 months to 6 years are more likely to ingest foreign objects because of their exploratory behavior of placing objects in their mouths, which can cause serious complications [4, 5]. Forrester [6] reported that 96% of patients were exposed at the patients’ residence, and 93% of the exposure routes were through ingestion among children aged ≤ 5 years. Nicotine toxicity and the exploratory behaviors of children are widely known among healthcare providers. However, it is not well known that some heated tobacco sticks may contain sharp foreign bodies that can cause hazardous complications by accidental ingestion.

**Fig. 3:**
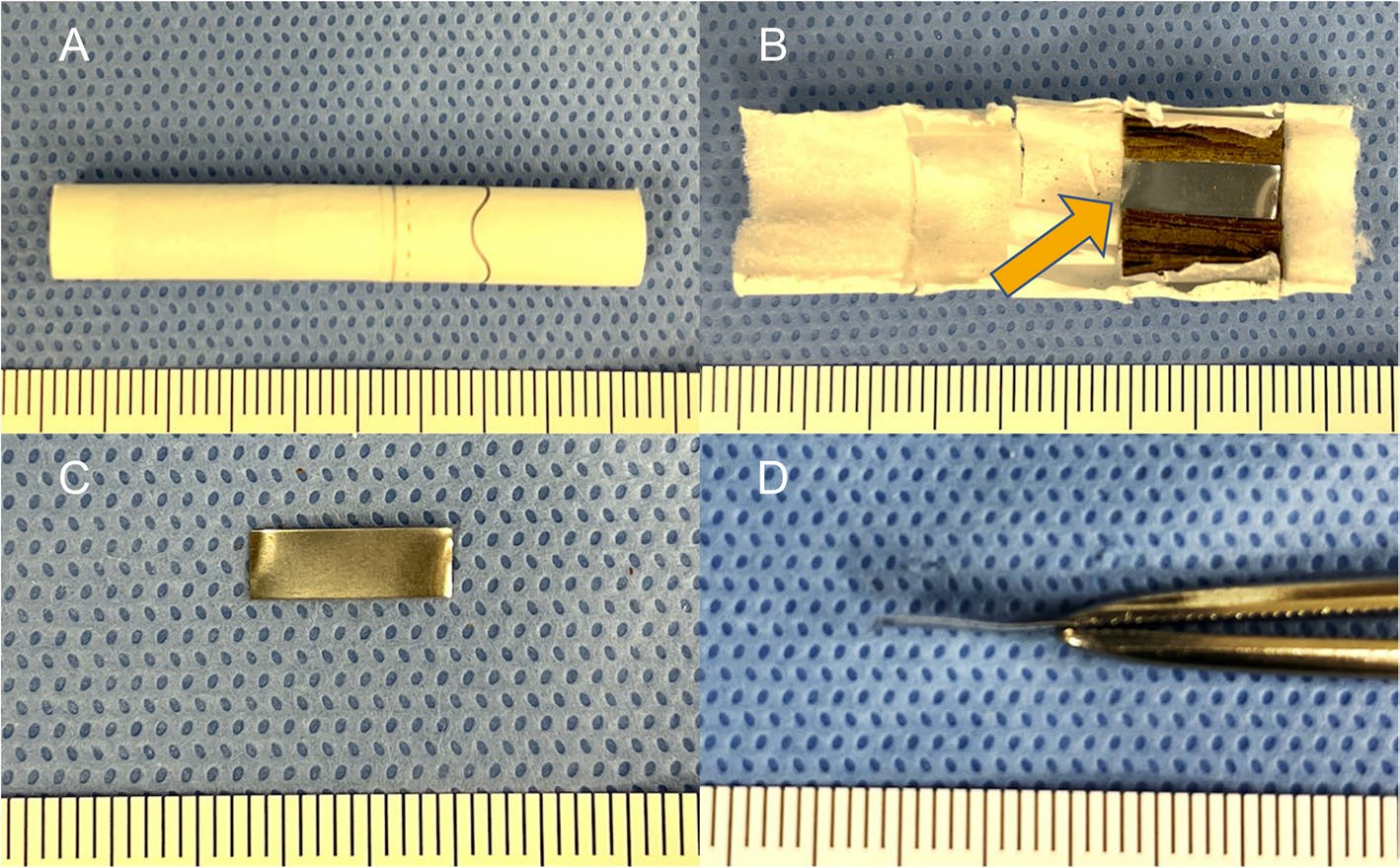
Images of a heated tobacco product. **A** A heated tobacco stick. **B** Contents of a heated tobacco stick (arrow). **C**, **D** A metallic susceptor

IQOS (I Quit Ordinary Smoking)™, manufactured by Philip Morris International, is one of the best-selling heated tobacco products worldwide. Parts of IQOS™, heated tobacco sticks called TEREA™ and SENTIA™, have been sold since 2021 in Japan and other countries [7]. These sticks contain a rectangular metallic susceptor **(**Fig. 3A, B**)** that is 12 mm long, 4 mm wide, and 0.06 mm thick **(**Fig. 3C, D**)**. This metallic object facilitates the heating and smoking of a cigarette without fire. The metallic susceptor has sharp corners, which can lead to bowel mucosal injury and gastrointestinal perforation if accidentally ingested. Ingestion of pointed foreign bodies in children is associated with high morbidity and mortality rates, and delayed diagnosis increases the risk of severe complications [8]. Endoscopic intervention is reportedly effective in 90% of patients who require retrieval of foreign bodies in the gastrointestinal tract, and this treatment is associated with few serious complications [9]. Therefore, clinicians should consider endoscopic removal of hazardous foreign bodies in the gastrointestinal tract whenever possible. In the case of sharp or pointed objects, several guidelines [10–12] recommend that endoscopic retrieval is desirable treatment even if the patient is asymptomatic. Although it has reported the majority of ingested foreign bodies pass through without incidents [11], the complication rates rise approximately 1% to between 15 and 35% due to ingestion of sharp or pointed objects [13]. Thus, we need to consider the indications cautiously based on the advantage and disadvantages. Several retrieval devices are available for the safe extraction of foreign bodies, for example grasping forceps, polypectomy snares, retrieval baskets, overtubes, and magnetic probes. In addition, removal of sharp foreign bodies using an endoscope with a cap might help to prevent esophageal and gastrointestinal injury [14]. Endoscopic intervention with a cap can facilitate partial capture of the pointed foreign bodies inside the cap, thus avoiding direct contact of the foreign body with the lumen wall [15]. We performed endoscopic extraction of a metallic susceptor by attaching a cap to the apex of the endoscope, and the extraction prevented serious complications. Ingested tobacco products are not usually detectable on X-ray films; however, TEREA™ and SENTIA™ can be detected because of their metallic susceptor. Therefore, if ingestion of a heated tobacco stick cannot be confirmed, X-ray examination is very useful for revealing evidence of a foreign body and its location.

Heated tobacco products are expected to spread globally, putting more children at risk of accidental ingestion and the need for treatment. It is essential to understand the current tobacco market situation and alert the public to the risk of these products because they may cause serious illness.

## Conclusions

We performed successful endoscopic removal of a metallic susceptor in the duodenum after ingestion of a heated tobacco called TEREA™. When children accidentally ingest heated tobacco sticks, clinicians must consider not only nicotine poisoning but also the possibility of a metallic foreign body within the digestive tract. We must caution the public about the risk of these products inducing serious complications and emphasize the necessity of early diagnosis and treatment.

## Data Availability

The patient’s data are not available because the personal information is protected.
